# Acute bilateral serous retinal detachments with spontaneous resolution in a 6-year-old boy

**DOI:** 10.3205/oc000164

**Published:** 2020-08-25

**Authors:** Sophie Van Camp, Steffi Vande Walle, Ingele Casteels, Julie Jacob, Cathérine Cassiman, Carine Wouters, Pieter-Paul Schauwvlieghe

**Affiliations:** 1 Department of Ophthalmology, University Hospitals Leuven, Belgium; 2 Department of Pediatrics, University Hospitals Leuven, Belgium

**Keywords:** serous retinal detachment, young child, acute exudative polymorphous vitelliform maculopathy

## Abstract

A healthy 6-year-old boy presented with acute bilateral vision loss, multiple serous retinal detachments between the vascular arcades and a thickened choroid. Spontaneous resolution occurred over several weeks.

We hypothesize that the clinical constellation in our patient is suggestive of acute exudative polymorphous vitelliform maculopathy (AEPVM) or might be an atypical presentation of Vogt-Koyanagi-Harada (VKH) disease. We propose that it was caused by an autoimmune-mediated activation of inflammatory cells at the level of the choroid, induced by an unknown trigger.

## Introduction

Acute exudative polymorphous vitelliform maculopathy (AEPVM) is a rare disorder, first described by Gass et al. in 1988 [1]. Patients usually present with bilateral blurred vision, often associated with headache. The typical fundoscopic findings consist of serous retinal detachments in both eyes, accompanied by scattered yellowish subretinal lesions in a honeycomb-like pattern. These lesions contain lipofuscin, which can be demonstrated on autofluorescence imaging. The clinical features can resolve spontaneously over the course of several months. Thus far no treatment has been found effective, nor has a single etiological hypothesis been proven.

## Case description

An otherwise healthy 6-year-old boy with no relevant medical history was referred to our department because of acute onset of bilateral blurry vision, metamorphopsia and scattered scotomas. He has a mother of Turkish descent and a Belgian father. The family history was negative for ophthalmological problems. There was no history of travelling to exotic regions. The patient had suffered from a cold a few weeks before the onset of symptoms.

**Figure 1:**
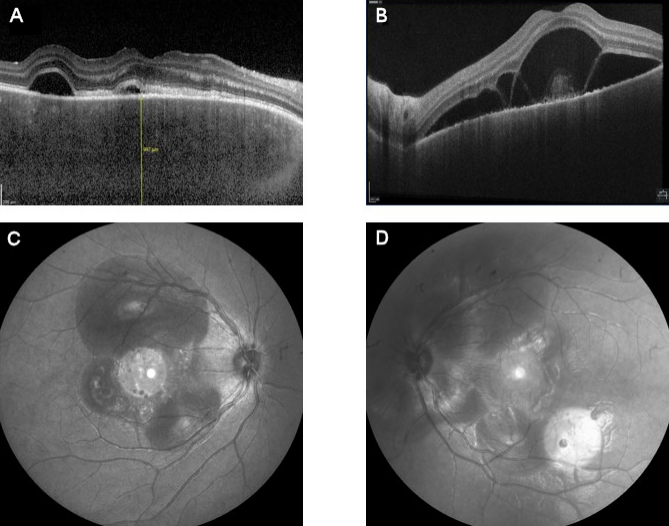
OCT and infrared images show multiple serofibrinous retinal detachments in the right (A, C) and left (B, D) eye and a thickened choroid measured with EDI-OCT (A).

**Figure 2:**
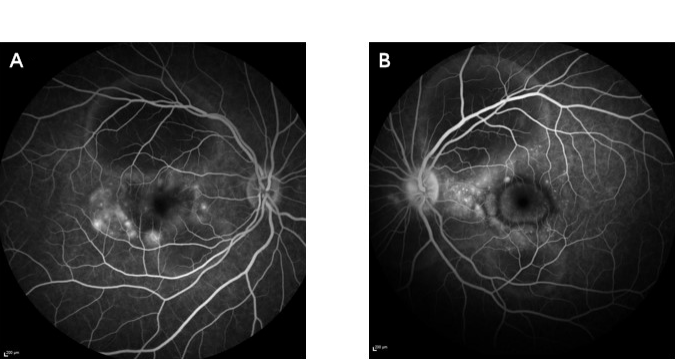
Late-phase fluorescein angiography shows tiny leakage points with limited leakage in the subretinal space (A, B) and discrete staining of the left optic disc (B).

We measured a best corrected visual acuity of Snellen 0.6 in the right eye and Snellen 0.16 in the left eye. Slitlamp biomicroscopy of the anterior segment showed no abnormalities and a normal intraocular pressure. Multiple large serofibrinous detachments with hyperreflective dots below the neurosensory retina were seen bilaterally on fundoscopic examination and optical coherence tomography (OCT) of the retina. These lesions were found to be hyperautofluorescent. A thickened choroid – estimated at 1000 µm – was detected on enhanced depth imaging optical coherence tomography (EDI-OCT) (Figure 1 (Fig. 1)). Infrared imaging showed serous detachments with a center of slightly increased intensity surrounded by decreased intensity (Figure 1 (Fig. 1)). Fluorescein angiography (FA) revealed small leakage points at the level of the serous detachments, limited staining of the optic disc in the left eye (without staining in the right eye) and masking effect due to neurosensory detachments (Figure 2 (Fig. 2)).

The patient was referred to our department of pediatrics, where a total clinical work-up did not show any evidence of systemic inflammation, infection or malignant diseases.

Serology for borreliosis, syphilis, toxoplasmosis, human immunodeficiency virus, herpes simplex, rubella, cytomegalovirus and Epstein-Barr virus was negative, in the absence of leukocytosis. A chest X-ray and magnetic resonance imaging (MRI) of the brain showed no abnormalities except for a thickened choroid in both eyes.

One week after initial presentation, spontaneous improvement of vision occurred with regression of the lesions on OCT. Minimal inflammation in the anterior chamber (without keratitic precipitates) and vitreous was seen and topical steroids were administered six times per day.

**Figure 3:**
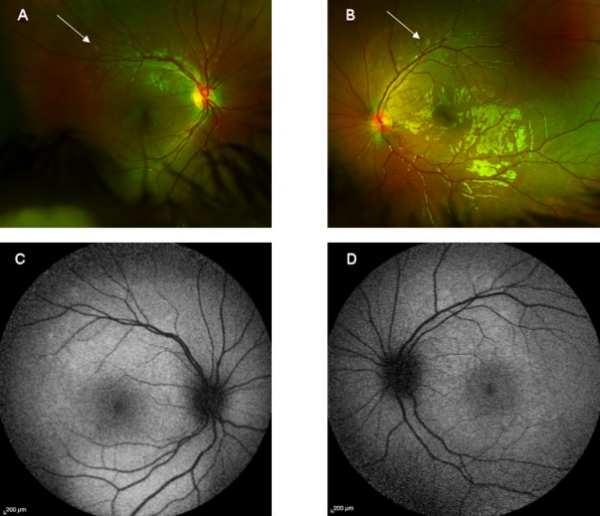
Fundoscopic images three weeks after initial presentation show multiple yellow deposits scattered around the vascular arcades (A and B, see arrows), with corresponding mild hyperautofluorescence (C, D).

Over the next two weeks, the serous retinal detachments resolved completely in the left eye and decreased markedly in the right eye, with distinct deposition of some yellowish, slightly hyperautofluorescent vitelliform material in the subretinal space (Figure 3 (Fig. 3)). Further improvement of visual acuity to Snellen 0.6 in both eyes (OU) occurred. Since intraocular inflammation was no longer present, topical steroids were tapered.

**Figure 4:**
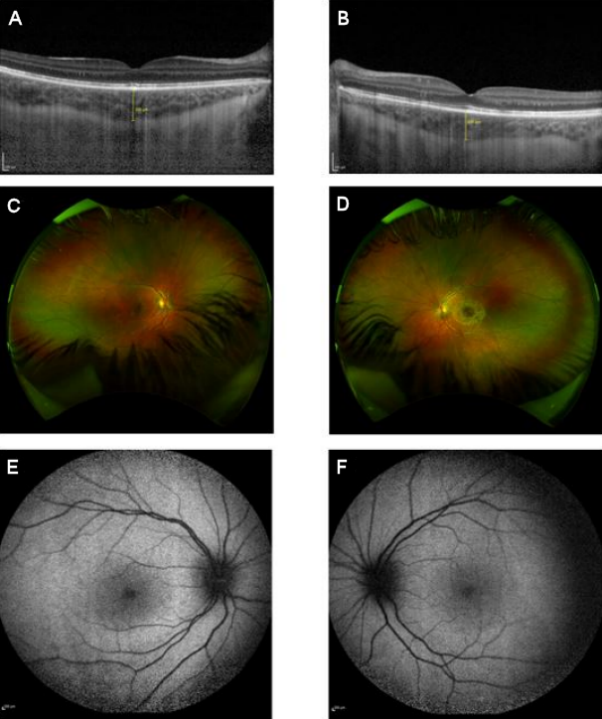
Three months after initial presentation, there was total resolution of the exudative retinal detachments in the right (A) and left (B) eye with normalization of choroidal thickness and an almost complete resorption of the vitelliform lesions (C, D). Autofluorescence was normal (E, F).

Three months after initial presentation, complete resolution of retinal lesions was noted with recovery of vision to Snellen 0.9 OU (Figure 4 (Fig. 4)).

## Discussion: differential diagnosis

### Acute exudative polymorphous vitelliform maculopathy (AEPVM)

AEPVM is a rare disorder, with symptoms of (sub)acute bilateral vision loss, frequently – but not always – preceded by headache [1], [2], [3]. Usually there are no or very few signs of intraocular inflammation. The fundoscopic findings are remarkable with bilateral multifocal central serous retinal detachments and round to oval white-yellowish lesions in a peculiar honeycomb-like pattern at the level of the retinal pigment epithelium, between and around the vascular arcades. These lesions do not occur in every patient, and they usually present after several months [2]. Fluorescein angiography shows early hyperfluorescence of the multifocal yellow lesions and staining in the late phase. Leakage of dye is minimal to absent. On autofluorescence imaging, lesions are hyperautofluorescent, suggesting the presence of lipofuscin [4]. Indocyanine green (ICG) angiography shows an affinity of the dye for these yellow lesions, exhibiting involvement of the choriocapillaris. One case reported choroidal thickening measured by EDI-OCT, with striking elevation in the acute phase [2], as seen in our patient. The serous retinal detachments typically resolve spontaneously over the course of weeks to months. This tends to occur faster if few or no yellow lesions are visible. These vitelliform lesions can gravitate and form a pseudo-hypopyon of yellow material at the level of the inferior macula, blocking fluorescence on FA. They can persist for years despite complete resolution of symptoms. This is the most typical presentation, although AEPVM can present with a more variable clinical course than initially described. We believe the lesions in our patient are very similar to the subtype ‘bleb-like lesions along vascular arcades’ of AEPVM [2].

The etiology of AEPVM remains unclear. Dysfunction of the retinal pigment epithelium (RPE) with decreased phagocytosis of secreted photoreceptor outer segments has been suggested, leading to secondary serous retinal detachment. This can cause precipitation and formation of yellow lesions, consisting of lipofuscin. OCT images have shown thickened outer segments, compatible with this hypothesis. The favorable response to corticosteroid therapy in some patients suggests an inflammatory base for this dysfunction [1].

A few cases have been reported following a viral [5] or bacterial infection [6], [7], [8]. These cases were thought to be based on an autoimmune-mediated activation of inflammatory cells (presumably T lymphocytes as in Vogt-Koyanagi-Harada disease) at the level of the choroid, leading to profound choroidal swelling. This could cause saturation of the choroid with subretinal accumulation of fluid rich in lipofuscin and exudative retinal detachment. A subsequent decrease in choroidal swelling – either spontaneously or by treatment with corticosteroids – can restore fluid absorption in the choroid with resolution of the retinal detachments. An incomplete clearance of lipofuscin causes yellow subretinal deposits. In our patient, a cold preceded the onset of symptoms, but we could not identify a specific causative micro-organism.

AEPVM has also been reported as a paraneoplastic retinopathy (e.g. metastatic melanoma, small cell lung carcinoma), where antibodies against an extraocular tumor supposedly cross-react with retinal or RPE antigens. Therefore, referral for systemic evaluation is always indicated [9], [10].

Treatment of AEPVM is controversial. In the first described cases of Gass et al., there was improvement after systemic prednisolone [1]. However, literature shows little evidence for the efficacy of corticosteroids; resolution of the lesions has been documented in many patients without any form of treatment.

One patient with AEPVM was treated in one eye with an intravitreal implant for sustained corticosteroid release [11]. There was no difference in the clinical course between both eyes, suggesting corticosteroids affect neither resolution of the lesions nor visual outcome. In another patient, intravenous corticosteroids led to a quick recovery of visual acuity, but the added value of corticosteroids on final visual acuity remains uncertain [12].

### Vogt-Koyanagi-Harada (VKH) disease

An atypical presentation of VKH disease was considered in our case, due to the bilateral presentation of serous retinal detachments in a child of Turkish descent. However, the typical vitelliform lesions in AEPVM do not occur in VKH disease and FA usually shows bilateral hot discs, which in our case was only present in the left eye (due to subretinal fluid – originating from the retinal detachments – in the peripapillary region, which led to staining of the optic disc). Since systemic features (auditory disturbances, skin changes or neurological symptoms) and panuveitis were absent, the diagnostic criteria were not met. Additionally, spontaneous recovery without aggressive treatment (i.e. systemic corticosteroids or other immunosuppressive agents) is not described in VKH disease [13], [14]. However, it must be mentioned that – although rare – VKH disease can be seen in children. There are only few case series describing VKH disease in children, in which a more aggressive course of disease and more frequent sight-threatening complications (e.g. cataract, glaucoma, retinal pigment epithelium atrophy) are noted [15], [16]. Thus, it is incorrect to assume that the disease has the same characteristics as in adult patients. Human leucocyte antigen (HLA) typing could help determine whether there is a genetic predisposition for VKH disease, but it cannot confirm the diagnosis. Therefore it was not tested in our case.

## Conclusion

A 6-year-old boy showed characteristics suggestive of the subtype ‘bleb-like lesions along the vascular arcades’ of AEPVM, but other features – such as a thickened choroid in the acute phase – resembled VKH disease, though as an atypical manifestation. To our knowledge, he is the youngest patient reported with the tentative diagnosis of AEPVM.

We hypothesize that the clinical picture was caused by a transient activation of inflammatory cells at the level of the choroid, resulting in choroidal swelling and subsequent accumulation of lipofuscin-rich fluid in the subretinal space. Spontaneous recovery of choroidal and RPE function occurred. Though asymptomatically, deposition of lipofuscin in the subretinal space, clinically appearing as yellow deposits, remained detectable for a limited period of time.

The underlying pathophysiological mechanism remains unclear. Infectious and paraneoplastic hypotheses have been suggested; we could not confirm any causative micro-organism in this case, nor withhold any arguments for RPE dysfunction or involvement of the choriocapillaris.

## Notes

### Informed consent

The parents of the patient gave consent to publish this case report.

### Competing interests

The authors declare that they have no competing interests.
